# High-resolution (1 km) Köppen-Geiger maps for 1901–2099 based on constrained CMIP6 projections

**DOI:** 10.1038/s41597-023-02549-6

**Published:** 2023-10-23

**Authors:** Hylke E. Beck, Tim R. McVicar, Noemi Vergopolan, Alexis Berg, Nicholas J. Lutsko, Ambroise Dufour, Zhenzhong Zeng, Xin Jiang, Albert I. J. M. van Dijk, Diego G. Miralles

**Affiliations:** 1 King Abdullah University of Science and Technology, Thuwal, Saudi Arabia; 2 CSIRO Environment, Canberra, ACT, Australia; 3 Australian Research Council Centre of Excellence for Climate Extremes, Canberra, ACT Australia; 4 Atmospheric and Ocean Sciences Program, Princeton University, Princeton, New Jersey USA; 5 NOAA Geophysical Fluid Dynamics Laboratory, Princeton, New Jersey USA; 6 University of Montreal, Montreal, Quebec Canada; 7 Scripps Institution of Oceanography, University of California, San Diego, La Jolla, California USA; 8 School of Environmental Science and Engineering, Southern University of Science and Technology, Shenzhen, China; 9 Fenner School of Environment & Society, The Australian National University, Canberra, Australia; 10 Hydro-Climate Extremes Lab (H-CEL), Ghent University, Ghent, Belgium

**Keywords:** Environmental health, Projection and prediction

## Abstract

We introduce Version 2 of our widely used 1-km Köppen-Geiger climate classification maps for historical and future climate conditions. The historical maps (encompassing 1901–1930, 1931–1960, 1961–1990, and 1991–2020) are based on high-resolution, observation-based climatologies, while the future maps (encompassing 2041–2070 and 2071–2099) are based on downscaled and bias-corrected climate projections for seven shared socio-economic pathways (SSPs). We evaluated 67 climate models from the Coupled Model Intercomparison Project phase 6 (CMIP6) and kept a subset of 42 with the most plausible CO_2_-induced warming rates. We estimate that from 1901–1930 to 1991–2020, approximately 5% of the global land surface (excluding Antarctica) transitioned to a different major Köppen-Geiger class. Furthermore, we project that from 1991–2020 to 2071–2099, 5% of the land surface will transition to a different major class under the low-emissions SSP1-2.6 scenario, 8% under the middle-of-the-road SSP2-4.5 scenario, and 13% under the high-emissions SSP5-8.5 scenario. The Köppen-Geiger maps, along with associated confidence estimates, underlying monthly air temperature and precipitation data, and sensitivity metrics for the CMIP6 models, can be accessed at www.gloh2o.org/koppen.

## Background & Summary

The Köppen-Geiger classification remains^1^, to this day, one of the most well-known and widely used climate classification systems. Developed in the late 19th century by Russian-German climatologist Wladimir Köppen^2,3^, and later refined by meteorologist Rudolf Geiger^4,5^, this classification divides global land climates into five major classes and 30 sub-classes, based on threshold values and seasonality of monthly air temperature and precipitation (Table 1). Drawing on his observation as a botanist that climatic conditions are a major driver of the global vegetation distribution, Köppen designed his classification to align with the major ecosystem types worldwide, with regions within the same class sharing broadly similar vegetation characteristics. As such, this classification system is useful for many ecological and ecohydrological applications and studies that depend on differences in climatic regimes, including climate change impact assessments^6–13^. More broadly, the Köppen-Geiger classification offers a way to aggregate complex land climate information into a meaningful indicator from both ecological and societal perspectives, and when coupled with climate change projections provides a useful schema to characterize the impacts on land surface condition in a comprehensive yet straightforward manner^14–19^.

**Table 1:** Overview of the Köppen-Geiger climate classes including the defining criteria.

Letter symbol				
1st	2nd	3rd	Description	Criterion^*a*^
A			Tropical	Not (B) & *T*_cold_ ≥ 18
	f		- Rainforest	*P*_dry_ ≥ 60
	m		- Monsoon	Not (Af) & *P*_dry_ ≥ 100-MAP/25
	w		- Savannah	Not (Af) & *P*_dry_ < 100-MAP/25
B			Arid	MAP < 10×*P*_threshold_
	W		- Desert	MAP < 5×*P*_threshold_
	S		- Steppe	MAP ≥ 5 × P_threshold_
		h	- Hot	MAT ≥ 18
		k	- Cold	MAT < 18
C			Temperate	Not (B) & *T*_hot_ > 10 & 0 < *T*_cold_ < 18
	s		- Dry summer	*P*_sdry_ < 40 & *P*_sdry_ < *P*_wwet_/3
	w		- Dry winter	*P*_wdry_ < *P*_swet_/10
	f		- Without dry season	Not (Cs) or (Cw)
		a	- Hot summer	*T*_hot_ ≥ 22
		b	- Warm summer	Not (a) & *T*_mon10_ ≥ 4
		c	- Cold summer	Not (a or b) & 1 ≤ T_mon10_ < 4
D			Cold	Not (B) & *T*_hot_ > 10 & *T*_cold_ ≤ 0
	s		- Dry summer	*P*_sdry_ < 40 & *P*_sdry_ < *P*_wwet_/3
	w		- Dry winter	*P*_wdry_ < *P*_swet_/10
	f		- Without dry season	Not (Ds) or (Dw)
		a	- Hot summer	*T*_hot_ ≥ 22
		b	- Warm summer	Not (a) & *T*_mon10_ ≥ 4
		c	- Cold summer	Not (a, b, or d)
		d	- Very cold winter	Not (a or b) & *T*_cold_ < −38
E			Polar	Not (B) & *T*_hot_ ≤ 10
	T		- Tundra	*T*_hot_ > 0
	F		- Frost	*T*_hot_ ≤ 0

Adapted from Peel *et al*.^39^.

^*a*^Variable definitions: MAT = mean annual air temperature (°C); *T*_cold_ = the air temperature of the coldest month (°C); *T*_hot_ = the air temperature of the warmest month (°C); *T*_mon10_ = the number of months with air temperature > 10 °C (unitless); MAP = mean annual precipitation (mm y^−1^); *P*_dry_ = precipitation in the driest month (mm month^−1^); *P*_sdry_ = precipitation in the driest month in summer (mm month^−1^); *P*_wdry_ = precipitation in the driest month in winter (mm month^−1^); *P*_swet_ = precipitation in the wettest month in summer (mm month^−1^); *P*_wwet_ = precipitation in the wettest month in winter (mm month^−1^); *P*_threshold_ = 2×MAT if > 70% of precipitation falls in winter, *P*_threshold_ = 2×MAT + 28 if > 70% of precipitation falls in summer, otherwise *P*_threshold_ = 2×MAT + 14. Summer (winter) is the six-month period that is warmer (colder) between April–September and October–March.

**Fig. 1:**
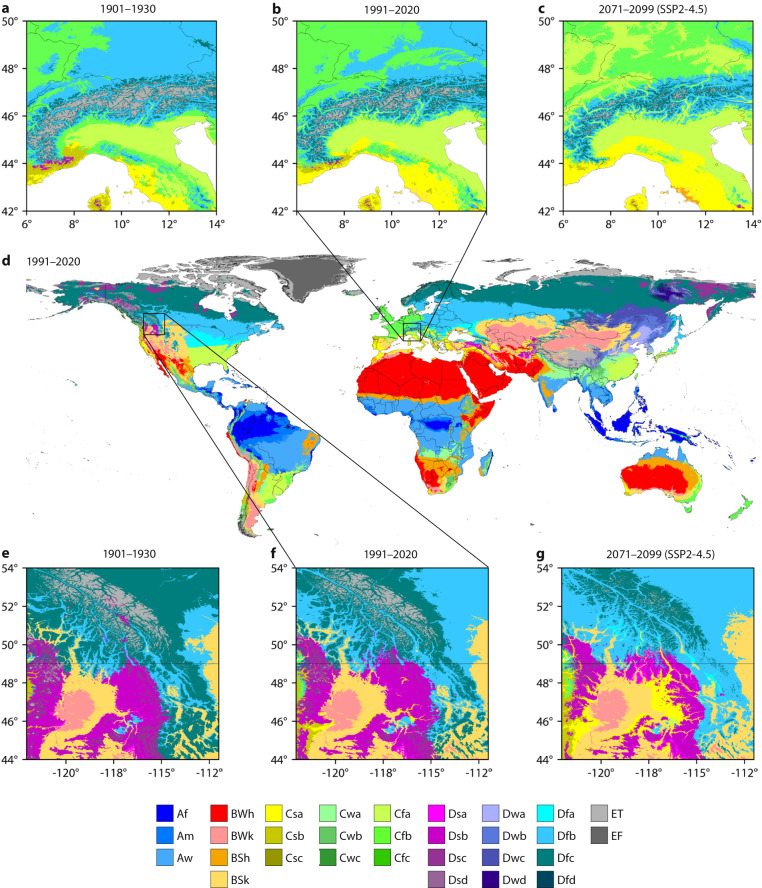
Köppen-Geiger classification for the European Alps (**a–c**), the global land surface (**d**), and the central Rocky Mountains (**e**–**g**) for 1901–1930 (**a,e**), 1991–2020 (**b,d,f**), and 2071–2099 (**c**,**g**) under the SSP2-4.5 scenario. In panels a–c the white areas are the Mediterranean Sea, with all seas and oceans being white in panel d.

Here, we introduce Version 2 of the 1-km Köppen-Geiger maps for historical and future climate conditions, with Version 1 published in 2018^18^ being widely used (e.g., cited 3075 times as of August 3, 2023, according to Google Scholar). The historical maps are derived from combinations of different high-resolution, observation-based climatologies for both V1 and V2, while in V2 the future maps are based on downscaled and bias-corrected climate projections from the Coupled Model Intercomparison Project phase 6 (CMIP6)^20^. V1 considered just one “worst case” future emissions scenario (Representative Concentration Pathway – RCP – 8.5)^21^ from CMIP5 and only one ensemble member (or variant) for each climate model. In contrast, V2 considers a broader range of seven future socio-economic scenarios (Shared Socio-economic Pathways – SSPs; SSP1-1.9, SSP1-2.6, SSP2-4.5, SSP3-7.0, SSP4-3.4, SSP4-6.0, and SSP5-8.5)^22–24^ and multiple ensemble members to reduce the uncertainty associated with internal climate variability^25,26^. Additionally, the new version covers six approximately 30-year periods (1901–1930, 1931–1960, 1961–1990, 1991–2020, 2041–2070, and 2071–2099), while V1 covered just two periods of different lengths (1980–2016 and 2071–2100). Figure 1 presents the newly derived Köppen-Geiger map for 1991–2020 and regional maps for the Alps (Europe) and the central Rocky Mountains (North America), illustrating the high level of detail. The classification accuracy, defined as the percentage of correctly classified classes using observations from meteorological stations worldwide as reference, ranged from 79.2% to 86.4% (Table 2).

**Table 2:** Classification accuracy of the Köppen-Geiger maps and mean confidence level of the correctly and incorrectly classified stations, respectively.

Period	Number of observations	Classification accuracy		Mean confidence level	
		30 sub-classes (%)	Five major classes (%)	Correctly classified (%)	Incorrectly classified (%)
1901–1930	3783	86.2	94.2	94.7	78.8
1931–1960	7667	86.4	94.7	94.1	75.2
1961–1990	16417	81.5	92.4	93.5	77.3
1991–2020	19643	79.2	91.7	93.4	78.7

**Fig. 2:**
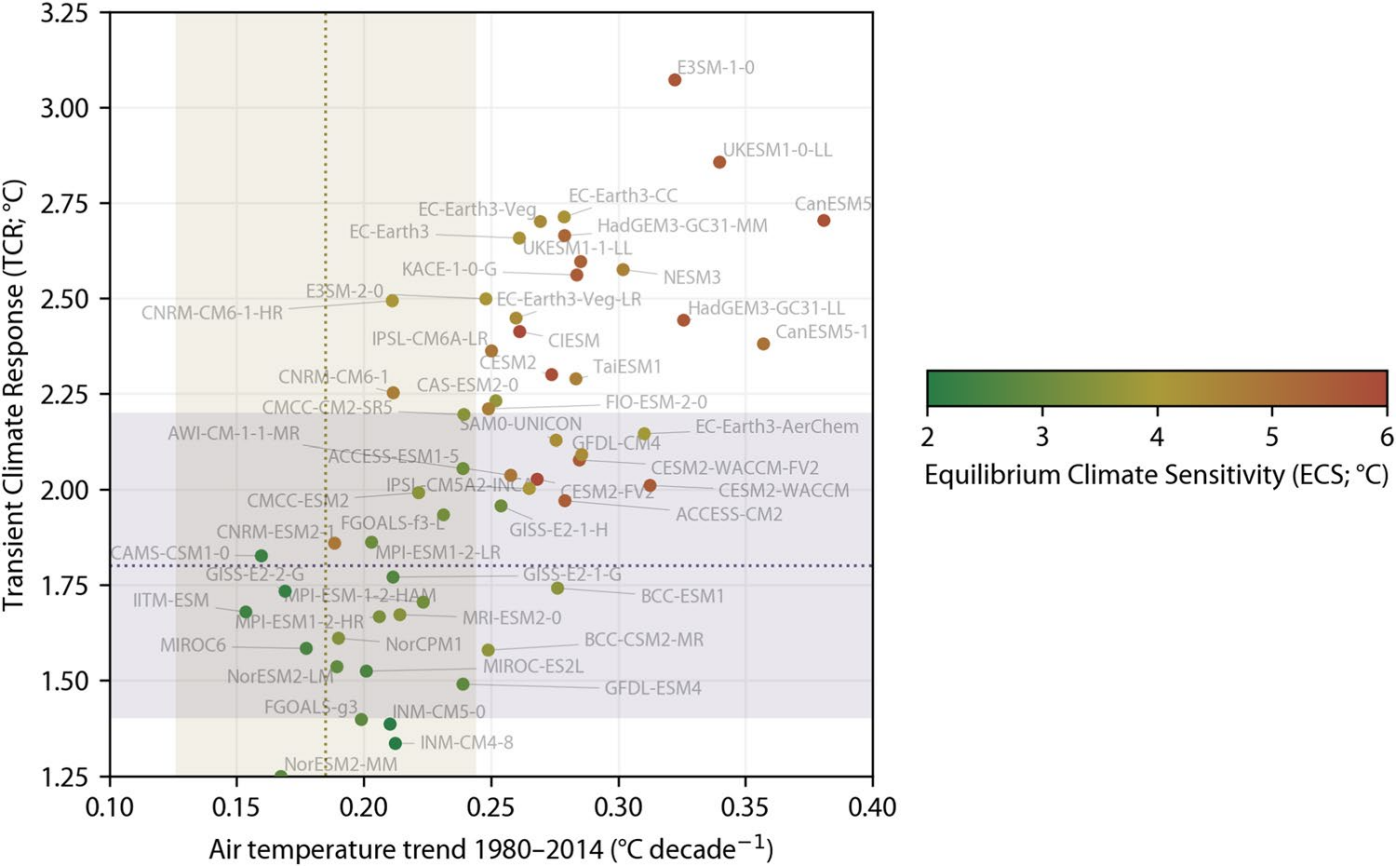
TCR and ECS values and global-mean air temperature trends for all CMIP6 models with the required data to compute all three statistics (*n* = 56). The yellow dotted line represents the best estimate historical trend with the yellow shaded area representing the likely range (68% confidence limit; see section “Constraining CMIP6 projections”). The gray dotted line represents the best estimate TCR from IPCC AR6^28^ with the gray shaded area indicating the likely range (66% confidence limit) also from IPCC AR6. Each circular marker represents a model. Models without sufficient data to calculate TCR or ECS values are not shown (*n* = 11). See Table 3 for historical trend, TCR, and ECS values (if available) for all models (*n* = 67).

**Fig. 3:**
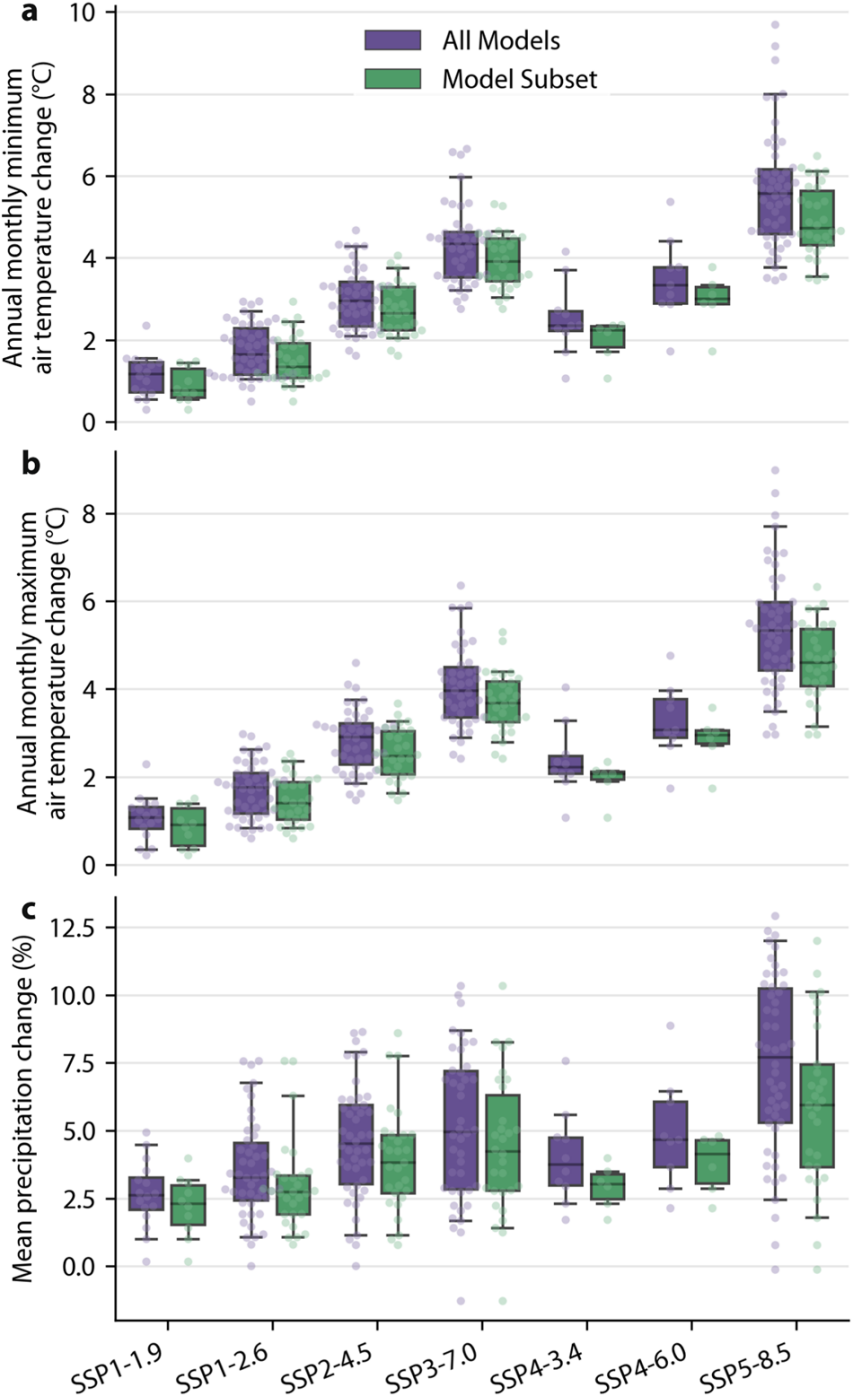
Mean change across the land surface in 2071–2099 (relative to 1991–2020) for different socio-economic scenarios (SSP1-1.9 to SSP5-8.5) based on ‘All Models’ (all CMIP6 models with sufficient data; *n* ≤ 67) and ‘Model Subset’ (screened model subset without less realistic models; *n* ≤ 42). Each circular marker represents a model. The number of models varies depending on the socio-economic scenario. The box shows the quartiles of the distribution, the whiskers indicate the 5th and 95th percentiles, while the black horizontal line in the box is the median.

**Fig. 4:**
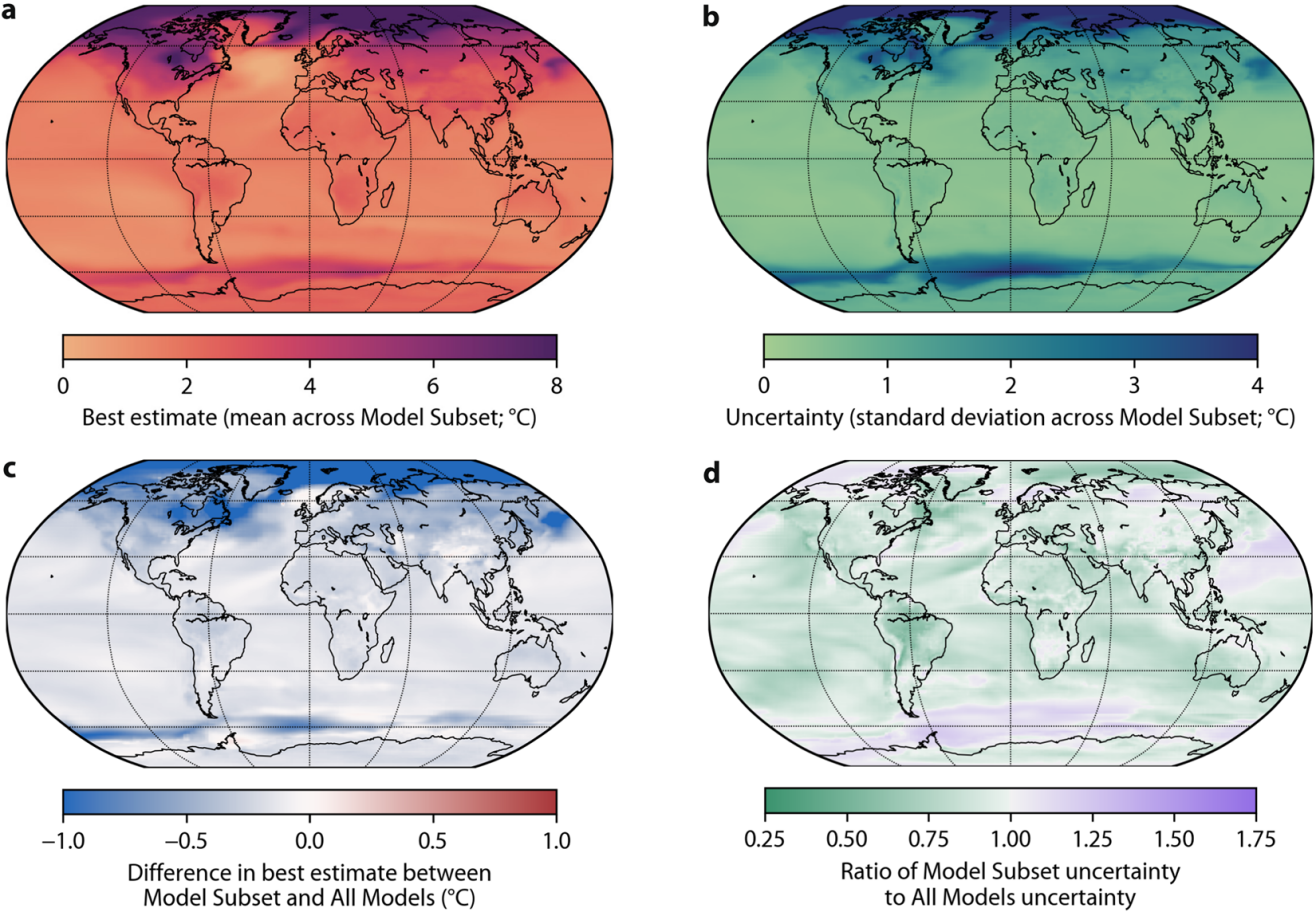
(**a**) Best estimate annual monthly minimum air temperature change in 2071–2099 (with respect to 1991–2020) under the SSP2-4.5 scenario (calculated as the mean across the Model Subset) with purple indicating a greater change. (**b**) Uncertainty corresponding to the best estimates (calculated as the standard deviation across the Model Subset) with dark blue indicating more uncertainty. (**c**) Difference in best estimate between Model Subset and All Models. Indicates how the model subsetting affects the best estimate with dark blue denoting greater changes. (**d**) Ratio of Model Subset uncertainty to All Models uncertainty. Indicates how the model subsetting affects the uncertainty with green denoting reduced uncertainty.

**Fig. 5:**
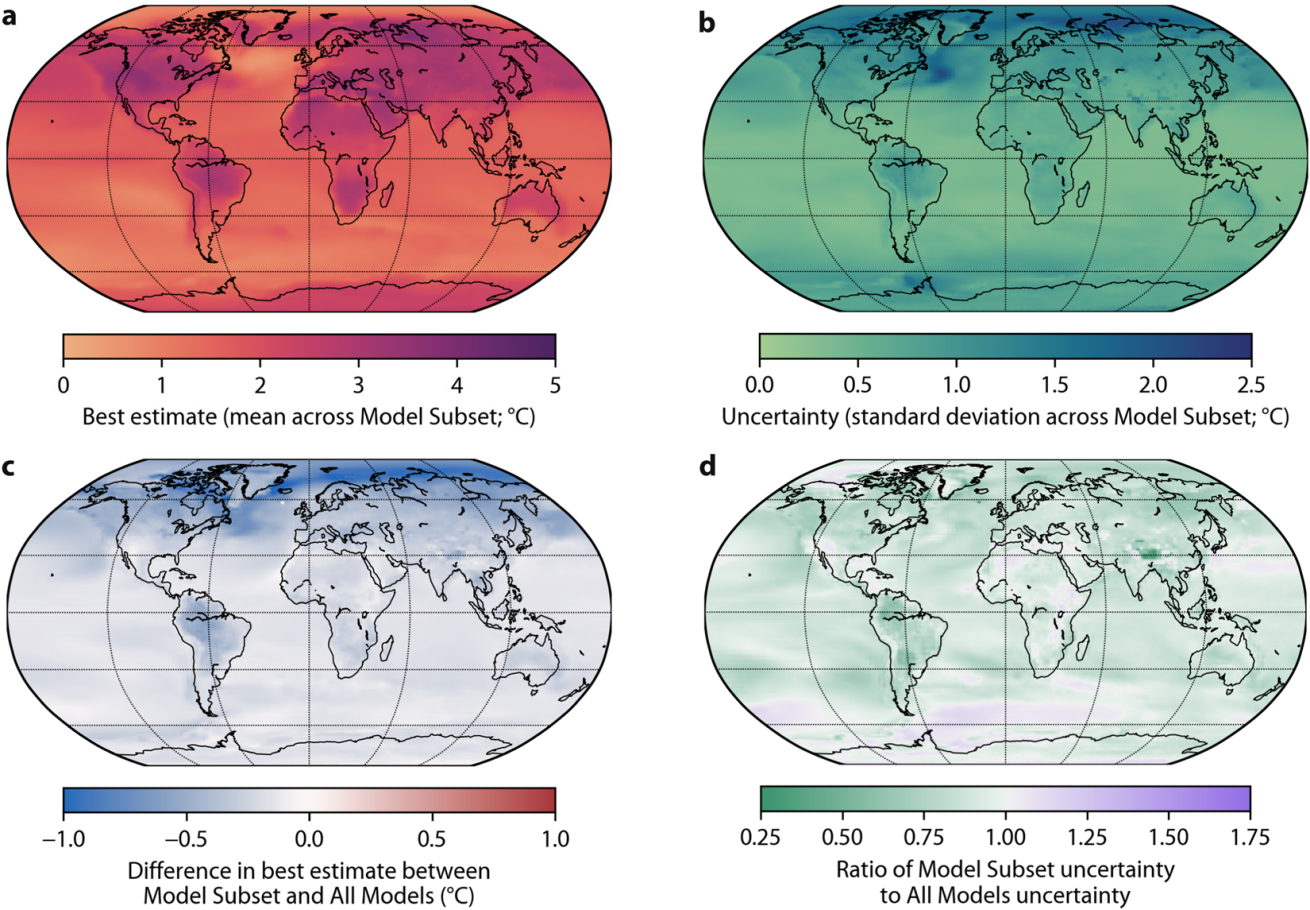
Same as Fig. 4 but for annual monthly maximum air temperature.

**Fig. 6:**
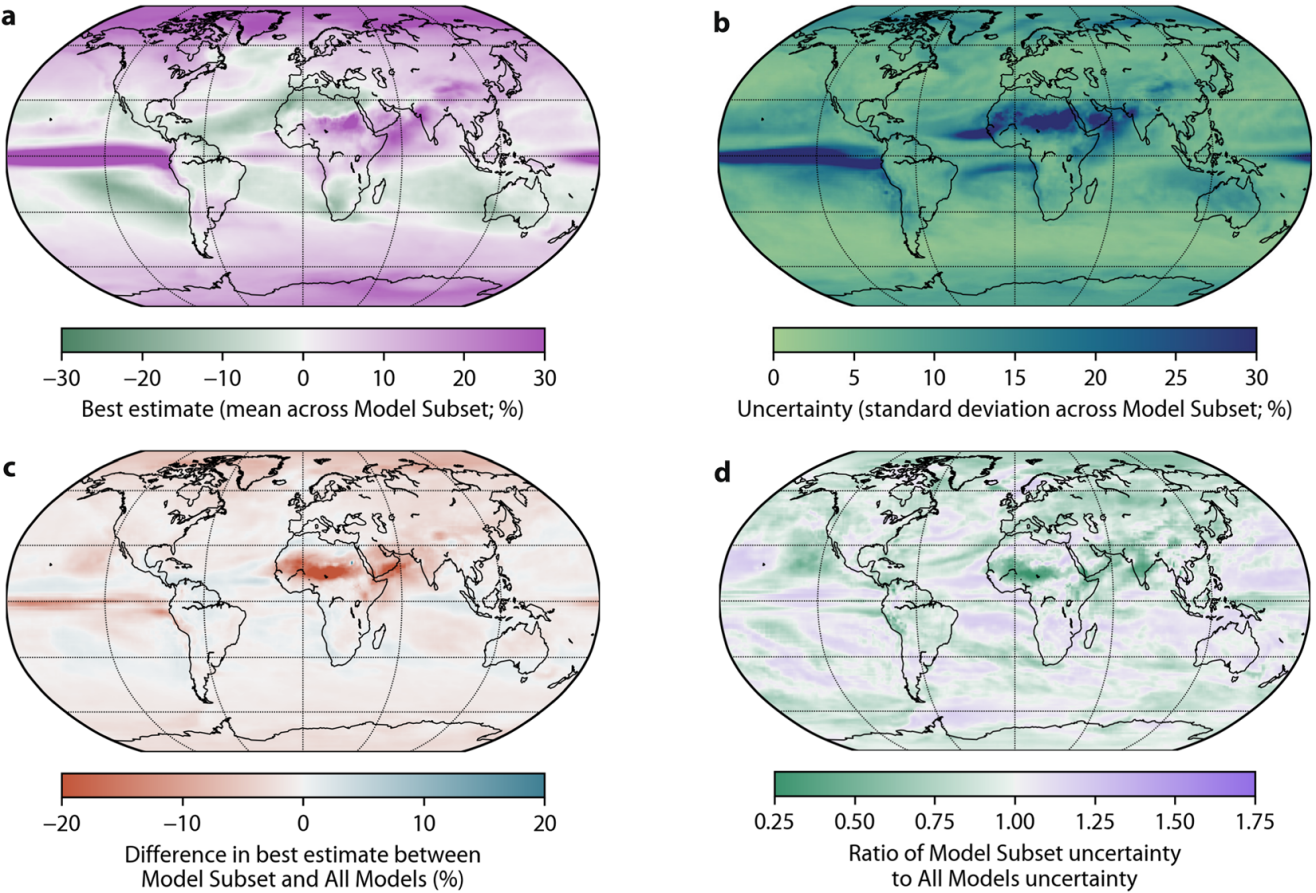
Same as Fig. 4 but for mean precipitation.

Recent studies found that a large number of CMIP6 climate models exhibit unrealistic CO_2_-induced warming rates^27–30^. To identify and exclude these models, we conducted a comprehensive assessment of CMIP6 climate models (see section “Constraining CMIP6 projections”). We evaluated 67 models based on: (i) historical air temperature trends (1980–2014)^27^; (ii) transient climate response (TCR)^31^; and (iii) equilibrium climate sensitivity (ECS)^32^ (Table 3 and Fig. 2). We found that 49 (73%) of the models fell outside the ‘likely’ range for at least one of these three sensitivity metrics. We discarded the 25 least realistic models and used the remaining subset of 42 models to derive the Köppen-Geiger maps and associated confidence maps. This approach ensured we maintained a statistically meaningful sample size for calculating averages and confidence levels for the different future socio-economic scenarios. By using this subset, the projected changes and uncertainties in annual monthly minimum and maximum air temperature and mean precipitation — the three climate variables used to distinguish between the five major Köppen-Geiger classes (Table 1) — are substantially reduced across the entire globe compared to using all models (Figs. 3–6). This result emphasizes the importance of careful model selection to guide critical climate-related decisions and investments^30^.

**Table 3:** The 67 CMIP6 models considered along with the corresponding number of ensemble members (or variants) used (up to 20 to conserve disk space), the global-mean air temperature trend for 1980–2014 (mean across ensemble members), the trend standard deviation (across ensemble members), the transient climate response (TCR; mean across ensemble members), the equilibrium climate sensitivity (ECS; mean across ensemble members), and whether the model was included in the final Model Subset (see section “Constraining CMIP6 projections”).

Model	Number of ensemble members	Mean trend (1980–2014; °C decade^−1^)	Trend standard deviation (1980–2014; °C decade^−1^)	Transient Climate Response (TCR; °C)	Equilibrium Climate Sensitivity (ECS; °C)	Included in ‘Model Subset’?
ACCESS-CM2	10	0.279	0.030	1.97	5.51	Yes
ACCESS-ESM1-5	20	0.258	0.045	2.04	4.91	Yes
AWI-CM-1-1-MR	5	0.239	—	2.05	3.14	Yes
AWI-ESM-1-1-LR	1	0.273	—	2.03	—	Yes
BCC-CSM2-MR	3	0.249	—	1.58	3.58	Yes
BCC-ESM1	3	0.276	—	1.74	3.52	Yes
CAMS-CSM1-0	3	0.160	—	1.83	2.31	Yes
CAS-ESM2-0	4	0.252	—	2.23	3.67	No
CESM2	11	0.274	0.034	2.30	5.86	No
CESM2-FV2	4	0.268	—	2.03	6.66	Yes
CESM2-WACCM	3	0.313	—	2.01	5.57	Yes
CESM2-WACCM-FV2	3	0.285	—	2.08	5.60	Yes
CIESM	3	0.261	—	2.41	6.26	No
CMCC-CM2-HR4	1	0.208	—	—	—	Yes
CMCC-CM2-SR5	11	0.239	0.053	2.20	3.50	Yes
CMCC-ESM2	1	0.222	—	1.99	3.53	Yes
CNRM-CM6-1	20	0.212	0.037	2.25	4.68	Yes
CNRM-CM6-1-HR	1	0.211	—	2.49	4.10	Yes
CNRM-ESM2-1	11	0.189	0.059	1.86	4.87	Yes
CanESM5	20	0.381	0.037	2.70	5.79	No
CanESM5-1	20	0.357	0.039	2.38	4.98	No
CanESM5-CanOE	3	0.358	—	2.63	—	No
E3SM-1-0	5	0.322	—	3.07	5.67	No
E3SM-1-1	1	0.309	—	—	—	No
E3SM-1-1-ECA	1	0.295	—	—	—	No
E3SM-2-0	5	0.248	—	2.50	4.12	No
EC-Earth3	20	0.261	0.070	2.66	4.21	No
EC-Earth3-AerChem	3	0.310	—	2.15	3.89	Yes
EC-Earth3-CC	10	0.279	0.068	2.71	4.19	No
EC-Earth3-Veg	9	0.269	—	2.70	4.43	No
EC-Earth3-Veg-LR	3	0.260	—	2.45	4.37	No
FGOALS-f3-L	3	0.231	—	1.93	3.17	Yes
FGOALS-g3	6	0.199	—	1.40	2.84	Yes
FIO-ESM-2-0	3	0.249	—	2.21	4.71	No
GFDL-CM4	1	0.286	—	2.09	4.36	Yes
GFDL-ESM4	3	0.239	—	1.49	2.86	Yes
GISS-E2-1-G	20	0.212	0.027	1.77	2.68	Yes
GISS-E2-1-G-CC	1	0.235	—	—	—	Yes
GISS-E2-1-H	20	0.254	0.042	1.96	3.12	Yes
GISS-E2-2-G	11	0.169	0.034	1.73	2.23	Yes
GISS-E2-2-H	5	0.220	—	1.87	—	Yes
HadGEM3-GC31-LL	5	0.326	—	2.44	5.65	No
HadGEM3-GC31-MM	4	0.279	—	2.66	5.30	No
IITM-ESM	1	0.154	—	1.68	2.37	Yes
INM-CM4-8	1	0.212	—	1.33	1.89	Yes
INM-CM5-0	10	0.210	0.028	1.39	2.05	Yes
IPSL-CM5A2-INCA	1	0.265	—	2.00	4.09	Yes
IPSL-CM6A-LR	20	0.250	0.044	2.36	4.90	No
IPSL-CM6A-LR-INCA	1	0.247	—	—	—	No
KACE-1-0-G	3	0.284	—	2.56	5.55	No
KIOST-ESM	1	0.271	—	—	4.63	No
MCM-UA-1-0	2	0.256	—	1.94	—	Yes
MIROC-ES2H	3	0.187	—	—	—	Yes
MIROC-ES2L	20	0.201	0.027	1.52	2.55	Yes
MIROC6	20	0.177	0.028	1.58	2.53	Yes
MPI-ESM-1-2-HAM	3	0.223	—	1.71	3.15	Yes
MPI-ESM1-2-HR	10	0.206	0.036	1.67	3.30	Yes
MPI-ESM1-2-LR	20	0.203	0.026	1.86	3.06	Yes
MRI-ESM2-0	12	0.214	0.027	1.67	3.45	Yes
NESM3	5	0.302	—	2.58	4.61	No
NorCPM1	20	0.190	0.026	1.61	3.38	Yes
NorESM2-LM	3	0.189	—	1.54	2.89	Yes
NorESM2-MM	3	0.167	—	1.25	2.78	Yes
SAM0-UNICON	1	0.276	—	2.13	4.28	Yes
TaiESM1	2	0.283	—	2.29	4.65	No
UKESM1-0-LL	19	0.340	0.042	2.86	5.54	No
UKESM1-1-LL	1	0.285	—	2.60	5.36	No

The trend standard deviation – used to estimate the uncertainty resulting from internal variability – is only provided for models with ≥10 ensemble members. The TCR values are only provided for models with data for both the 1pctCO2 and piControl experiments, while the ECS values are only provided for models with data for both the abrupt-4xCO2 and piControl experiments.

**Fig. 7:**
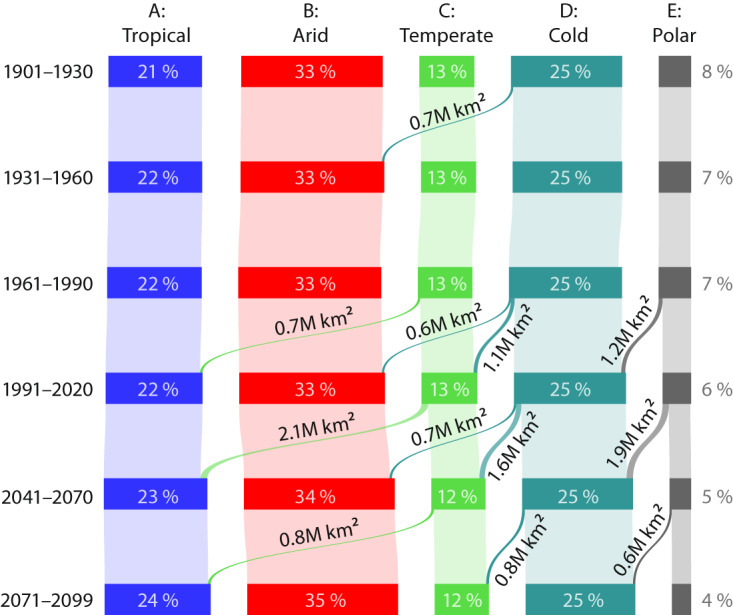
The global distribution and transitions of the five major Köppen-Geiger classes from 1901 to 2099 under the SSP2-4.5 scenario. Only transitions covering a surface area greater than 0.6 million km^2^ (roughly the area of Ukraine) are shown to avoid clutter. The capital ‘M’ stands for million. The percentages are expressed as a proportion of the global land surface area excluding Antarctica (137 million km^2^).

The updated Köppen-Geiger maps provide a comprehensive assessment of the spatio-temporal distribution of climate classes across the global land surface from 1901 to 2099 (Figs. 1, 7). Based on the revised maps, we estimate that from 1901–1930 to 1991–2020, approximately 5% of the global land surface (excluding Antarctica) transitioned to a different major Köppen-Geiger class. From 1991–2020 to 2071–2099, 5% of the land surface is projected to transition to a different major class under the low-emissions SSP1-2.6 scenario, 8% under the middle-of-the-road SSP2-4.5 scenario, and 13% under the high-emissions SSP5-8.5 scenario. Under SSP2-4.5, the global land surface area (excluding Antarctica) with favorable climatic conditions for tropical, arid, temperate, cold, and polar vegetation is expected to undergo net changes of + 8%, + 4%, −3%, −2%, and −33%, respectively, from 1991–2020 to 2071–2099. Furthermore, we estimate that 2.6 million km^2^ (roughly the area of Argentina, the world’s eighth largest country) of the global land surface will transition from polar (E) to cold (D) between 1991–2020 and 2071–2099 under scenario SSP2-4.5, while 2.4 million km^2^ will transition from cold (D) to temperate (C), 1.1 million km^2^ will transition from cold (D) to arid (B), and 2.8 million km^2^ will transition from temperate (C) to tropical (A).

Our results align reasonably well with previous assessments^8,12,17,33–35^, with differences being expected as we considered a wider range of socio-economic scenarios, a larger number of climate models, and multiple ensemble members from each model. In addition, we excluded less realistic models and produced our maps at a higher resolution (1 km). It should be noted that changes in biomes may not necessarily occur in concert with changes in Köppen-Geiger classes due to other factors affecting vegetation not included in the classification such as local geography, soil type, increased CO_2_ concentrations, nutrient availability, wildfires, invasive species disruptions, human interventions (including deforestation, urbanization, and agriculture), as well as the inherent transition times of ecosystems^36–38^.

## Methods

### Köppen-Geiger climate classification

Table 1 presents the Köppen-Geiger climate classification used herein, which is identical to that used in Version 1^18^ as well as several other studies^39,40^. This classification largely reflects Köppen’s seminal publication from 1936^3^, with three key modifications. Firstly, temperate (C) and cold (D) climates are distinguished using a 0 °C threshold, as opposed to Köppen’s 3 °C, as per Russell’s (1931)^41^ recommendation. Secondly, arid (B) subclimates, W (desert), and S (steppe) are identified based on whether 70% of the precipitation falls in summer or winter. Thirdly, the subclimates s (dry summer) and w (dry winter) within the C and D climates are made mutually exclusive by classifying a summer as dry (s) if more precipitation falls in winter, and a winter as dry (w) if the opposite is true. The tropical (A), temperate (C), cold (D), and polar (E) climates are by definition mutually exclusive but can overlap with the arid (B) class. To resolve this, the B climate type is prioritized over all others.

### Historical Köppen-Geiger maps

Historical Köppen-Geiger classification maps for 1901–1930, 1931–1960, 1961–1990, and 1991–2020 were derived from seven high-resolution, topographically-corrected, observation-based climatic datasets: three for near-surface air temperature and four for precipitation. We used multiple datasets due to the inherent uncertainty in determining the most accurate one^42,43^. Moreover, using multiple datasets typically enhances accuracy by reducing the impacts of errors in individual datasets and enables the quantification of uncertainty from the spread across these datasets^44,45^. The air temperature climatic datasets used were: (i) WorldClim V2^46^ (covering 1970–2000); (ii) Climatologies at High resolution for the Earth’s Land Surface Areas (CHELSA) V1.2^47^ (1979–2013); and (iii) CHELSA V2.1^47^ (1981–2010). The precipitation climatic datasets used were: (i) WorldClim V2^46^ (1970–2000); (ii) CHELSA V1.2 (1979–2013); (iii) CHELSA V2.1^47^ (1981–2010); and (iv) Climate Hazards Group’s Precipitation Climatology (CHPclim) V1^48^ (1980–2009). All these datasets have a 0.01° resolution, except CHPclim V1, which has a 0.05° resolution. To ensure consistency with the other datasets, CHPclim V1 was resampled to 0.01° using bilinear interpolation.

The climatic datasets differ in their temporal coverage (e.g., CHPclim covers 1980–2009, whereas WorldClim V2 covers 1970–2000). However, we require climatic data for the four selected historical periods (1901–1930, 1931–1960, 1961–1990, and 1991–2020). To adjust the temporal coverage of the climatic datasets, we calculated monthly climate change offsets (for air temperature) or factors (for precipitation) between the historical period in question and the temporal coverage of the climatic dataset. To compute the air temperature offsets, we used Climatic Research Unit (CRU) Time Series (TS) V4.07^49^ air temperature data, which has a monthly 0.5° resolution covering 1901–2022. To compute the precipitation factors, we used the Global Precipitation Climatology Centre (GPCC) Full Data Reanalysis (FDR) V2022^50,51^, which has a monthly 0.25° resolution covering 1891–2020. We downscaled these offsets and factors to a 0.01° resolution using bilinear interpolation and then adjusted the climatic datasets for each month, either by adding the offsets (for air temperature) or multiplying by the derived factors (for precipitation).

We subsequently generated Köppen-Geiger maps at 0.01° resolution for each historical period and combination of adjusted air temperature and precipitation climatic datasets. Next, we created for each historical period a final Köppen-Geiger map from the ensemble of 12 (4×3) maps by selecting the mode for each grid-cell (i.e., the most common class). A corresponding confidence map was also generated by dividing the frequency of occurrence of the most common class by the ensemble size and expressing the result as a percentage. For example, if the most common class for a particular grid-cell is Csa, and it was assigned nine times out of 12, the resulting confidence level would be 100×9/12 = 75%. This confidence level indicates the degree of trust we place in our final climate classification. Confidence levels are generally lower near the borders between climate classes.

### Constraining CMIP6 projections

Several climate models included in CMIP6 have unrealistically hot projections^27,29,30^, which is largely due to the representation of clouds and their response to increased CO_2_ concentrations^52,53^. To identify and exclude these models, we calculated three metrics to evaluate their sensitivity to changes in atmospheric CO_2_ concentrations: (i) the global-mean historical air temperature trend for 1980–2014^27^; (ii) the transient climate response (TCR), which estimates the global-mean warming around the time when CO_2_ doubles^31^; and (iii) the equilibrium climate sensitivity (ECS), which estimates the eventual steady-state global-mean warming at double CO_2_^32,54^. Although the Köppen-Geiger classification incorporates both air temperature and precipitation, we only focused on sensitivity metrics related to air temperature for two main reasons. First, there is no discernible historical trend in precipitation observations, as the global mean greenhouse gas effect on precipitation has been offset by the global mean aerosol effect^55,56^. Second, historical temperature observations are more robust and widespread than precipitation observations^46,57^.

#### Simulated historical global-mean air temperature trends

We calculated trends in global-mean near-surface air temperature for 1980–2014 for each CMIP6 model using data from the historical experiment^20^, downloaded from the Earth System Grid Federation (ESGF) platform (https://esgf-node.llnl.gov; Table 3 and Fig. 2). During this period, the impact of aerosol forcing was relatively small, and the warming was primarily driven by changes in greenhouse gas forcing^27,58^. The end of this period, 2014, corresponds to the end of the historical experiment. For each model, we averaged all available ensemble members, up to 20, to conserve disk space. This reduces the impact of internal climate variability, that is, the inherently unpredictable variation in climate not resulting from changes in greenhouse gas concentrations but from the chaotic nature of the system^25,26^. Internal variability can be a confounding factor when comparing observed and simulated air temperature trends, as it can cause differences between the two that are not due to changes in anthropogenic forcing^25,26^.

#### Observed historical global-mean air temperature trend

We estimated the observed historical global-mean near-surface air temperature trend for 1980–2014 using gridded anomalies from the Hadley Centre/Climatic Research Unit Temperature (HadCRUT) analysis V5.0.1.0^59^ (monthly 0.5° resolution). Unlike the CRU TS dataset, which only covers the land surface, the HadCRUT dataset is based on both weather station and sea surface temperature data and covers the entire globe. HadCRUT includes data from 1850 to the present, and any missing data earlier in the record was filled with the global mean. The HadCRUT dataset comprises 200 ensemble members sampling the uncertainty arising from: (i) basic measurement uncertainty; (ii) correction for changes in instrumentation and measurement practices; and (iii) the estimation of gridded fields from a sparse distribution of observations. We calculated the global-mean trend for 1980–2014 for each ensemble member. The mean trend across the ensemble (termed *μ*_obs_) was 0.1851 °C decade^−1^ and the standard deviation across the ensemble was 0.0038 °C decade^−1^ (hereafter referred to as the structural uncertainty or *σ*_struct_). The use of sea surface temperature data rather than near-surface air temperature data over oceans in HadCRUT results in an underestimation of the actual trend, due to the delayed response of the oceans to global warming^60^. This underestimation (hereafter referred to as the blending bias or *ε*_blend_) was previously estimated at 0.014 °C decade^−1^ ^27^.

We used near-surface air temperature data from the CMIP6 historical experiment to quantify the uncertainty resulting from internal climate variability. For all CMIP6 models with ≥10 ensemble members, we calculated trends in global-mean air temperature for 1980–2014 for each ensemble member (each representing a different realization of the internal variability). We used up to 20 ensemble members, to conserve disk space. We then calculated the standard deviation of the trends across the ensemble members for each model, providing an estimate of the uncertainty associated with internal variability based on a single model. In total, there were 22 models with ≥10 ensemble members, and the mean uncertainty resulting from internal variability across these models (termed *σ*_internal_) was 0.0391 °C decade^−1^ (standard deviation 0.0129 °C decade^−1^; Table 3). This estimate aligns closely with a previously reported value of 0.038 °C decade^−1^ ^27^. The likely global-mean air temperature trend range (68% confidence interval) was subsequently determined at 0.130–0.241 °C decade^−1^ according to:

1 $${T}_{{\rm{range}}}=\left({\mu }_{{\rm{obs}}}+{\varepsilon }_{{\rm{blend}}}\right)\pm \sqrt{{\sigma }_{{\rm{struct}}}^{2}+2{\sigma }_{{\rm{internal}}}^{2}},$$

where *T*_range_ is the likely global-mean air temperature trend range, *μ*_obs_ is the observed ensemble-mean global-mean air temperature trend, *ε*_blend_ is the blending bias, *σ*_struct_ is the structural uncertainty, and *σ*_internal_ is the uncertainty due to internal variability (all in °C decade^−1^). The term $\sqrt{{\sigma }_{{\rm{struct}}}^{2}+2{\sigma }_{{\rm{internal}}}^{2}}$ represents the combined uncertainty arising from both the observations and internal variability. The uncertainty associated with internal variability is doubled since both the observations and simulations represent different realizations of the internal variability. For models with multiple ensemble members, *T*_range_ may be somewhat conservative, as the uncertainty due to internal variability is averaged out to a certain degree.

#### Transient climate response (TCR)

The TCR quantifies the change in global-mean near-surface air temperature in the year when CO_2_ concentrations have doubled after continuously increasing by 1% every year^31,61,62^ (Table 3 and Fig. 2). The TCR represents the initial warming that occurs after a rapid increase in CO_2_ concentration before the climate system has fully adjusted. It was estimated for each CMIP6 model following Intergovernmental Panel on Climate Change (IPCC)’s Fifth Assessment Report (AR5)^63^ by calculating the global-mean near-surface air temperature difference between the 1% CO_2_ increase experiment (1pctCO2^20^) and the pre-industrial control run (piControl^20^) averaged over a 20-year period centered on year 70, at which CO_2_ concentrations have doubled. To minimize the impact of internal climate variability on our TCR estimates^26^, we calculated the mean TCR across all available ensemble members, up to 20, to conserve disk space. These ensemble members are the same as used in section “Observed historical global-mean air temperature trend”. We could not estimate the TCR for some models due to a lack of data for the 1pctCO2 or piControl experiments. The likely TCR range (66% confidence interval) was determined to be 1.4–2.2 °C in the IPCC Sixth Assessment Report (AR6)^28^ based on multiple lines of evidence from paleoclimate, historical air temperature observations, and ocean heat content.

#### Equilibrium climate sensitivity (ECS)

The ECS quantifies the long-term global-mean near-surface air temperature change after CO_2_ concentrations have doubled and the climate system has reached a state of equilibrium where the temperature has stabilized^32,54,61^ (Table 3 and Fig. 2). The ECS was estimated for the CMIP6 models following Gregory *et al*.^32^ from the regression of global annual mean top of the atmosphere net downward radiative flux anomaly (*N*; W m^−2^) against global annual mean near-surface air temperature anomaly (Δ*T*; K; Table 3 and Fig. 2). *N* and Δ*T* were calculated from the difference between the abrupt 4×CO_2_ experiment (abrupt-4xCO2^20^) and the pre-industrial control run (piControl^20^). The Δ*T* at the intersection of the regression line with *N* = 0 W m^−2^ represents the change in air temperature when the climate system has reached equilibrium. This Δ*T* is divided by two to obtain the air temperature response per doubling of CO_2_ concentration, as per the definition of the ECS. While Gregory *et al*.^32^ used years 1–150 years for the regression, we used only years 21–150 to obtain ECS estimates which are slightly higher, but in better agreement with slab ocean models and long simulations (≥800 years)^64^. Just as we did for the TCR, we calculated, for each model, the mean ECS across all available ensemble members (up to 20), to minimize the impact of internal climate variability on our estimates. The likely ECS range (66% confidence interval) was determined to be 2.5–4.0 °C in the IPCC Sixth Assessment Report (AR6)^28^, which is similar to the range of 2.6–4.1 °C reported in a recent review^54^.

#### Model assessment

We found that only 28 (42%) of the 67 CMIP6 models had historical air temperature trends within the likely range, 33 (55%) of the 60 models which provided data from 1pctCO2 simulations had TCR values within the likely range (1.4–2.2 °C), and 21 (37%) of the 57 models which provided data from abrupt-4xCO2 simulations had ECS values within the likely range (2.5–4.0 °C; Table 3 and Fig. 2). Just 18 (27%) of the 67 models had all available metrics within the likely range. Notwithstanding some differences related to the specific methods selected to estimate TCR^65^ and ECS^64^, these results accord with previous climate model sensitivity assessments^61,62,64^. These findings emphasize that uncritically using the full ensemble of models can be misleading and should not guide important real-world decisions^30^.

To derive the future Köppen-Geiger maps, we excluded models based solely on historical air temperature trends and TCR estimates. The TCR is more relevant to this study than the ECS, as it represents the expected amount of climate change in the coming decades. Moreover, Huusko *et al*.^66^ showed that under the SSP2-4.5 scenario, the TCR is a better predictor of regional warming throughout the 21st century. From the 60 models with both historical air temperature trends and TCR estimates, we excluded the 21 with both estimates outside the likely range. From the 7 models without TCR estimates, we excluded the 4 with historical air temperature trends outside the likely range. The remaining subset of 42 models is herein denoted as the ‘Model Subset’, and the full complement of 67 models as ‘All Models’ (Table 3). Since only a few models had projections for all seven socio-economic scenarios, we opted for relatively conservative exclusion criteria, ensuring a statistically meaningful sample of models were used for each scenario to calculate averages and confidence levels. For SSP1-1.9, this resulted in 10 models; for SSP1-2.6, 29 models; for SSP2-4.5, 29 models; for SSP3-7.0, 30 models; for SSP4-3.4, 6 models; for SSP4-6.0, 6 models; and for SSP5-8.5, 29 models.

#### The impact of excluding less realistic models

Figure 3 presents projected global-mean changes in three climate indices at the end of the 21st century using the Model Subset and All Models for all seven socio-economic scenarios. The three climate indices (mean precipitation, annual monthly minimum air temperature, and annual monthly maximum air temperature) are used in the Köppen-Geiger classification to distinguish between the five major classes (A–E; Table 1). For all socio-economic scenarios and indices, the magnitude of the median change (i.e., the best estimate) and the interquartile range (i.e., the uncertainty) across the models is less for Model Subset than for All Models. This once again highlights the importance of excluding less realistic models to avoid exaggerated climate change projections and reduce uncertainty^27,29,30^. Even though the Model Subset selection did not involve assessing precipitation trends, the magnitude of the best estimate change and uncertainty were also reduced for mean precipitation when compared to All Models (Fig. 3a). This is attributed to the influence of air temperature on evaporation and the water-holding capacity of the atmosphere, which subsequently influence the processes governing precipitation frequency and intensity^67–69^. It should be noted that for scenarios with a limited number of models, such as SSP1-1.9, SSP4-3.4, and SSP4-6.0, the reduction in uncertainty might be somewhat overestimated. This is because measures of dispersion, like the interquartile range, derived from small samples tend to underestimate the true dispersion of the population^70^.

Figures 4–6 present global maps of projected changes in the three climate indices at the end of the 21st century based on the Model Subset and All Models. We only present results for the middle-of-the-road SSP2-4.5 scenario^71^. Annual monthly minimum and maximum air temperature are projected to increase across the entire globe, particularly minimum air temperature at higher latitudes (Figs. 4a, 5a). Worldwide, the projected change and the uncertainty in annual monthly minimum and maximum air temperature are substantially lower using the Model Subset than using All Models (Figs. 4c,d, 5c,d). Mean precipitation is projected to decrease over Central America, the Mediterranean, Southern Africa and Australia, and increase elsewhere over land (Fig. 6a). The projected change in mean precipitation is generally of lesser magnitude using the Model Subset than using All Models (Fig. 6c), consistent with the results for air temperature indices (Figs. 4c, 5c)^67,69^. The uncertainty in mean precipitation projections is less for the Model Subset than for All Models over most of the globe, except for Australia and parts of the US, northern Europe, Africa, and the Middle East (Fig. 6d).

### Future Köppen-Geiger maps

We generated Köppen-Geiger maps at 0.01° resolution for the 2041–2070 and 2071–2099 periods and for seven socio-economic scenarios: SSP1-1.9, SSP1-2.6, SSP2-4.5, SSP3-7.0, SSP4-3.4, SSP4-6.0, and SSP5-8.5^22,23^. The maps were produced using monthly near-surface air temperature and precipitation projections from CMIP6^20^ downloaded from the ESGF platform (https://esgf-node.llnl.gov). We only used the models included in Model Subset (see section “Constraining CMIP6 projections”). The historical data from 1850–2014 were combined with scenario data from 2015–2100. We averaged over all available ensemble members (up to 20) to reduce the uncertainty associated with internal climate variability^25,26^. For models with more than 20 ensemble members, we sorted the ensemble members in their “natural” order (i.e., multi-digit numbers were treated as if they were a single character) and selected the first 20.

We used the so-called delta-change approach^72,73^ to increase the spatial resolution (or downscale) the climate model data, enabling the generation of the high-resolution future Köppen-Geiger maps. This simple yet effective approach superimposes the climate change signal, derived from the models, onto the high-resolution climatic maps. We implemented the approach in four steps. Firstly, we derived monthly reference air temperature and precipitation climatologies (0.01° resolution) for 1991–2020 by averaging the ensemble of temporally-adjusted, high-resolution climatic maps (see section “Historical Köppen-Geiger maps”). Secondly, for each scenario, climate model, future period, and month, we calculated climate change offsets (for air temperature) or factors (for precipitation) between 1991–2020 and the future period. These offsets or factors were then resampled from the native model resolution to 0.01° using bilinear interpolation. Thirdly, we derived downscaled future monthly air temperature and precipitation climatologies, by adding the offsets (for air temperature) or multiplying by the factors (for precipitation). Fourthly, and finally, we generated future Köppen-Geiger maps (0.01° resolution) from the downscaled future monthly air temperature and precipitation climatologies.

For each scenario, we derived a final Köppen-Geiger map from the ensemble of maps (representing different climate models) by selecting the mode (the most common class) for each grid-cell. Similar to the historical maps, we also generated corresponding confidence maps. The confidence level was quantified by dividing the frequency of occurrence of the most common class by the ensemble size and expressing the result as a percentage.

## Data Records

The historical and future Köppen-Geiger classification maps, associated confidence maps, and underpinning monthly near-surface air temperature and precipitation climatologies can all be downloaded from figshare^1^ and www.gloh2o.org/koppen. The data are available at four spatial resolutions: 0.01°, 0.1°, 0.5°, and 1°; which correspond to approximately 1 km, 11 km, 56 km, and 111 km at the equator, respectively. The Köppen-Geiger maps were resampled from 0.01° to the coarser resolutions by majority resampling, while the confidence maps and climatologies were resampled by averaging. The future maps and climatologies are available for seven socio-economic scenarios (SSP1-1.9, SSP1-2.6, SSP2-4.5, SSP3-7.0, SSP4-3.4, SSP4-6.0, and SSP5-8.5). For convenience, the data are organized into six ZIP archives:

1. koppen_geiger_tif.zip (90 MB): This is the only archive needed for the vast majority of users. It contains GeoTIFF files with Köppen-Geiger maps in different resolutions for various periods and socio-economic scenarios. For instance, the file 2071_2099/ssp585/koppen_geiger_0p01.tif contains the Köppen-Geiger map for the period 2071–2099 under scenario SSP5-8.5 at a resolution of 0.01°. GeoTIFF files can be easily viewed using commonly used Geographic Information System (GIS) software, such as QGIS and ArcGIS. The archive also includes a legend file, legend.txt, which links the numeric values in the maps to the Köppen-Geiger climate symbols and provides the color scheme we adopted from Peel *et al*.^39^ for displaying the maps.
2. koppen_geiger_nc.zip (761 MB): This archive contains the same Köppen-Geiger maps as the preceding archive, except in NetCDF format, under the variable kg_class. The confidence maps are also included, under the variable kg_confidence. Similar to the previous archive, the maps are provided for different periods and scenarios, and at various resolutions. For instance, the variable kg_class in the file 2041_2070/ssp245/koppen_geiger_0p5.nc provides the Köppen-Geiger map for the period 2041–2070 under scenario SSP2-4.5 at a resolution of 0.5°. The archive also contains the previously described legend.txt file.
3. climate_data_0p01.zip (69 GB): This archive contains NetCDF files with temperature climatologies (under the variable air_temperature in °C) and precipitation climatologies (under the variable precipitation in mm month^−1^) at a resolution of 0.01°. The variables have dimensions of 18000×36000×12. Both the mean and the standard deviation across the model ensemble are included; the mean serves as our best estimate, while the standard deviation represents the associated uncertainty. Similar to the preceding archives, the climatologies are provided for various periods and scenarios. For instance, the variable precipitation in the file 1991_2020/ensemble_mean_0p01.nc provides the ensemble-mean precipitation climatology for the period 1991–2020 at a resolution of 0.01°.
4. climate_data_0p1.zip (1.3 GB): This archive is identical to the previous one, but provides the data at a coarser resolution of 0.1°.
5. climate_data_0p5.zip (83 MB): This archive is identical to the previous one, but provides the data at a further coarsened resolution of 0.5°.
6. climate_data_1p0.zip (25 MB): This archive is identical to the previous one, but provides the data at the coarsest resolution of 1°.

## Technical Validation

### Station data

The new 0.01° historical Köppen-Geiger maps were validated using observations from 170 699 meteorological stations worldwide from the following ten sources: (i) the Global Historical Climatology Network-Daily (GHCN-D) dataset^74^ (ftp.ncdc.noaa.gov/pub/data/ghcn/daily/; 122 728 stations), (ii) the Global Summary Of the Day (GSOD) dataset (https://data.noaa.gov; 25 571 stations), (iii) the Latin American Climate Assessment & Dataset (LACA&D) dataset (http://lacad.ciifen.org; 231 stations), (iv) the Chile Climate Data Library (www.climatedatalibrary.cl; 716 stations), (v) the FLUXNET2015 dataset^75^ (https://fluxnet.org; 206 stations), and national datasets for (vi) Bolivia (57 stations), (vii) Brazil (12 410 stations), (viii) Mexico (5398 stations), (ix) Peru (255 stations), and (x) Iran (3127 stations).

To eliminate long sequences of erroneous zero precipitation often present in GSOD time series^76,77^, we applied a central moving mean with a one-year window. We only assigned a value if at least half a year’s worth of values were present and retained only those observations with a non-zero coincident moving mean. Similarly, to eliminate long sequences of erroneous non-zero precipitation in GSOD time series, we calculated a central moving minimum with a one-year window. A value was only assigned if at least half a year’s worth of data was present, and we only retained observations with a coincident moving minimum of zero.

For each historical period and station, we calculated monthly mean air temperature and precipitation time series (discarding months with < 20 daily values), and subsequently monthly climatologies by averaging the monthly means (if ≥10 values were present). For each historical period, we only discarded stations without climatic averages for all twelve months.

### Classification accuracy

Table 2 presents the classification accuracy (defined as the percentage of correct classifications) of the historical Köppen-Geiger maps, calculated using observations from meteorological stations. The number of stations grew from 3783 in the earliest historical period (1901–1930) to 19 643 in the latest one (1991–2020), reflecting the substantial expansion of weather monitoring networks during the 20th century^78^. The classification accuracy ranged from 79.2% to 86.4% for the 30-sub classes, similar to the accuracy of 80.0% reported by Beck *et al*.^18^. Interestingly, the accuracy for 1991–2020 was slightly lower than that for 1901–1930 (79.2% versus 86.2%). This difference can likely be attributed to the manifestation of climate change in recent decades, which makes pinpointing a single class more challenging. Furthermore, recent decades have seen a higher proportion of stations in less developed areas, where the data quality might not be as high. As expected, the classification accuracy was higher for the five major classes than for the 30 sub-classes, ranging from 91.7% to 94.7%.

Our validation may slightly overestimate the true accuracy of the maps for two reasons. First, some of the station data used for validation was also used to produce the high-resolution, topographically-corrected climatologies and the GPCC FDR and CRU TS datasets (see section “Historical Köppen-Geiger maps”). This was unavoidable due to a lack of freely available, independent station data. Second, the majority of stations used for validation are situated in populated, relatively flat, mid-latitude areas, characterized by frontal weather. Observations from these areas tend to be quite accurate. In contrast, tropical, mountainous, and high-latitude regions are more influenced by convective weather, orographic precipitation, and snowfall, respectively. These regions are underrepresented in the validation data, and their observations are often less accurate^46,79–81^.

The value of the confidence maps associated with the Köppen-Geiger maps was assessed by comparing the mean confidence levels for incorrectly and correctly classified stations for each historical period. For 1991–2020, the mean confidence level was 93.4% for correctly classified stations and 78.7% for incorrectly classified stations (Table 2). The mean confidence level was thus lower for the incorrectly classified stations, suggesting that the confidence maps provide a useful indication of the classification accuracy. The mean confidence levels were similar for the other historical periods.

## Usage Notes

The newly derived Köppen-Geiger maps provide a unique, high-resolution view of the evolution of climate classes across the global land surface from 1901 to 2099 for seven socio-economic scenarios (Fig. 1). We conducted a comprehensive assessment of CMIP6 climate models and excluded models with less realistic CO_2_-induced warming rates (Figs. 2, 3). The encouraging classification accuracy results suggest that our maps are reliable (Table 2). However, there are six important caveats when using the maps:

1. It should not be assumed that future changes in the Köppen-Geiger classification will directly result in a change in a specific biome. Changes are likely to occur gradually through the process of succession, in which plants and animals colonize and modify an ecosystem over time^38^. Moreover, the Köppen-Geiger classification depends solely on climate. Other important factors include local geography, soil type, rising CO_2_ concentrations, grazing pressure, wildfires, invasive species disruptions, and human interventions such as deforestation, urbanization, and agriculture^36,37^. Hence, the future Köppen-Geiger classification should first and foremost be interpreted from a climate perspective.
2. The confidence levels associated with the historical Köppen-Geiger maps only take into account the uncertainty in the high-resolution, station-based climatologies used to create them (see section “Historical Köppen-Geiger maps”). They do not consider the uncertainty in the CRU TS^49^ and GPCC FDR^50,51^ datasets that were used to adjust the maps to cover a different time span. Hence, the confidence intervals in the historical maps may be overestimated, particularly in the early 20th century, when the uncertainty in the CRU TS and GPCC FDR datasets is likely to be higher. This is an unavoidable limitation, as the CRU TS and GPCC FDR datasets do not quantify the uncertainty arising from both interpolation and measurement errors.
3. Many of the CMIP6 climate models used to create the future Köppen-Geiger maps have a relatively coarse spatial resolution (1° or approximately 100 km; see section “Future Köppen-Geiger maps”). This coarse resolution limits their ability to accurately capture small-scale processes and represent complex or heterogeneous regions, such as coastlines, islands, and mountainous areas^82^. In these regions, the confidence levels might be overly optimistic, and the Köppen-Geiger maps should be interpreted with more caution. For instance, many models are unable to simulate the amplified warming resulting from the positive feedback of snow and ice melt in mountainous regions^83^. Consequently, our Köppen-Geiger maps might underestimate potential changes in these areas.
4. The confidence levels of the future Köppen-Geiger maps represent the uncertainty stemming from differences among the climate models for a specific socio-economic scenario, rather than the overall uncertainty including both model and scenario uncertainty^84^ (see section “Future Köppen-Geiger maps”). This overall uncertainty would be higher and the corresponding confidence levels lower, particularly at the end of the 21st century when scenario uncertainty begins to dominate model uncertainty^85^. Calculating the overall uncertainty is not straightforward, as this would require assigning probabilities to the different scenarios. Note that the model uncertainty also includes some uncertainty due to internal variability^25,26^, although the latter has been greatly reduced herein due to the use of multiple ensemble members for several of the models (Table 3).
5. Our approach to select a subset of climate models with realistic CO_2_-induced warming rates (referred to as ‘Model Subset’) for generating the future Köppen-Geiger maps is not without limitations (see section “Constraining CMIP6 projections”). Firstly, while the ‘likely’ climate sensitivity ranges we used for model selection reflect the prevailing scientific consensus^28,54^, they remain a subject of ongoing debate^86,87^, and future refinements to these ranges are expected. Secondly, to ensure we had an adequate number of models for each socio-economic scenario, we included models with either the historical near-surface air temperature trend or the TCR outside the likely range. As a result, Model Subset might still slightly overestimate future warming. Thirdly, and lastly, we neither excluded nor downweighted models from the same genealogy, that is, different versions or variants of models with a common origin. Consequently, the biases or errors associated with these similar models may be overrepresented in Model Subset^88–90^.
6. Global-mean warming changes for 2071–2099 (with respect to 1850–1900, also known as the ‘pre-industrial’ period) are projected to be 1.51 °C for SSP1-1.9, 1.97 °C for SSP1-2.6, 2.75 °C for SSP2-4.5, 3.59 °C for SSP3-7.0, 2.33 °C for SSP4-3.4, 2.98 °C for SSP4-6.0, and 4.21 °C for SSP5-8.5 based on the Model Subset (which excludes less realistic models). These warming levels were calculated by summing the change for 1991–2020 (with respect to 1850–1900) from HadCRUT^59^ and the mean change for 2071–2099 (with respect to 1991–2020) from the climate models. Hence, only for “sustainability” scenarios SSP1-1.9 and SSP1-2.6^91^ does warming stay below the 2 °C threshold set by the Paris Agreement, which was signed in 2015 by 200 countries^92^.

## Data Availability

The new Köppen-Geiger classifications have been produced using Python version 3.10. The code can be accessed at https://github.com/hylken/Koppen-Geiger_maps and is licensed under the GNU General Public License v3.0.
